# A genome-wide analysis of Cas9 binding specificity using ChIP-seq and targeted sequence capture

**DOI:** 10.1093/nar/gkv137

**Published:** 2015-02-20

**Authors:** Henriette O'Geen, Isabelle M. Henry, Mital S. Bhakta, Joshua F. Meckler, David J. Segal

**Affiliations:** 1 Genome Center and Department of Biochemistry and Molecular Medicine, University of California, Davis, CA 95616, USA; 2 Department of Plant Biology and Genome Center, University of California, Davis, CA 95616, USA

## Abstract

Clustered regularly interspaced short palindromic repeat (CRISPR) RNA-guided nucleases have gathered considerable excitement as a tool for genome engineering. However, questions remain about the specificity of target site recognition. Cleavage specificity is typically evaluated by low throughput assays (T7 endonuclease I assay, target amplification followed by high-throughput sequencing), which are limited to a subset of potential off-target sites. Here, we used ChIP-seq to examine genome-wide CRISPR binding specificity at gRNA-specific and gRNA-independent sites for two guide RNAs. RNA-guided Cas9 binding was highly specific to the target site while off-target binding occurred at much lower intensities. Cas9-bound regions were highly enriched in NGG sites, a sequence required for target site recognition by *Streptococcus pyogenes* Cas9. To determine the relationship between Cas9 binding and endonuclease activity, we applied targeted sequence capture, which allowed us to survey 1200 genomic loci simultaneously including potential off-target sites identified by ChIP-seq and by computational prediction. A high frequency of indels was observed at both target sites and one off-target site, while no cleavage activity could be detected at other ChIP-bound regions. Our results confirm the high-specificity of CRISPR endonucleases and demonstrate that sequence capture can be used as a high-throughput genome-wide approach to identify off-target activity.

## INTRODUCTION

Targeted genome engineering by nucleases has enabled researchers to alter genetic content in a variety of cell types and organisms. In particular, the RNA-guided Cas9 endonuclease, adapted from the clustered regularly interspaced short palindromic repeat (CRISPR) system of *Streptococcus pyogenes*, has emerged as the universal tool of choice for advancing biological research as well as the potential for therapy of genetic diseases (1–3). Cas9 is guided to genomic loci by a guide-RNA (gRNA) containing 20 nt complementary to the genomic target site, which is immediately upstream of a protospacer adjacent motif (PAM) site. The PAM site consists of the three-nucleotide sequence NGG and is a requirement for Cas9 binding to its target region (4,5).

Wild-type Cas9 nuclease acts by introducing double strand breaks at the DNA target site that are either repaired by NHEJ (non homologous end joining) or HR (homologous recombination). HR occurs at much lower frequencies, which can be increased by providing donor DNA template. The default pathway of NHEJ causes insertion or deletion mutations (indels) (4,6). The occurrence of indels is commonly used to determine RNA-guided cleavage efficiency of Cas9 nuclease. Single amino acid mutations in the nuclease domains convert Cas9 nuclease into a nickase, while introducing two amino acid changes (D10A and H840A) result in a nuclease-inactive DNA binding protein (dCas9) (4). dCas9 can be fused to heterologous effector domains to regulate transcription (7–9). In addition, dCas9 could be fused to domains that regulate the epigenetic landscape at endogenous loci. This strategy has shown potential for zinc finger and transcription activator-like effector (TALE) DNA binding proteins (10–13). The versatility and ease of use make CRISPR/Cas9 a powerful tool for genome editing and gene regulation, but our understanding of binding specificity and target recognition remains limited.

Most studies have identified indels introduced by Cas9 nucleases at off-target sites that share sequence similarity to the target site (14–19). All studies were in concurrence about the importance of the PAM site and concluded that mismatches in the 5′ region of the target site were much better tolerated than in the PAM-adjacent sequence, also referred to as the seed region. The seed region has been defined as the sequence of 6 to 12 bp immediately upstream of to the PAM site. However, the search for off-target events was limited to predicted off-target sites, and thus subject to potential biases based on the quality of the predictions. Although four studies concluded that Cas9:gRNA nucleases were very specific (14,15,18,19), two studies found strong off-target activity (16,17). Off-target effects were observed at endogenous loci that differ by one to six positions from the actual target sequence. It has become clear that off-target effects vary greatly by site and it has proven difficult to identify a pattern to predict binding specificity.

Target site recognition and DNA binding are required prior to CRISPR/Cas9 activity. ChIP-seq (Chromatin Immunoprecipitation followed by high-throughput sequencing) is a powerful tool to identify genome-wide binding sites in an unbiased manner. Recently, ChIP-seq studies have generated genome-wide maps of dCas9 binding (20–22). The number of off-target sites varied with the amount of expressed dCas9 protein and was also dependent on gRNA target sequence. Different gRNA sequences resulted in highly variable numbers of identified off-target binding sites, ranging from 26 to 5957 (22) and from 13 to 1281 (21). Among the 9594 gRNA-specific peaks (22), a subset of 295 (3%) dCas9-bound regions was chosen for validation of off-target cleavage activity using targeted polymerase chain reaction (PCR) and sequencing. Cas9 cleavage activity was only detected at one of the chosen ChIP-bound sites.

Off-target site predictions based on sequence similarity or genome-wide binding analysis by ChIP-seq have not been reliable predictors of off-target cleavage by CRISPR/Cas9 (1,14–18,21–23). Validation of off-target cleavage activity has largely been based on a selection of genomic off-targets that were PCR amplified and then screened for the presence of indels using either high-throughput sequencing or Sanger sequencing. These cleavage assays from genome-wide Cas9 binding studies were still limited to a subset of potential off-target sites and a high-throughput method that allows simultaneous validation of a large number of potential off-target sites would greatly benefit the field.

Whole genome sequencing (WGS) of Cas9 treated cells can identify indels genome-wide, but faces its own challenges and limitations. This has recently been demonstrated in induced Pluripotent Stem Cells (iPSCs). WGS of individual iPSC clones reveals a large number of indels in the genome that are not the result of Cas9 activity, but are frequently a consequence of clonal variation or technical artifacts (24–26). As WGS produces an extensive list of indels, only indels occurring in proximity to a potential off-target site or PAM were considered to be the result of Cas9 activity. All three studies were in concordance that Cas9 cleavage exhibits low off-target activity. However, it is worth mentioning that 60× coverage identified only one indel at the target site (25). Sequencing of a single human genome (∼3 Gb) to 60× coverage requires ∼4.5 lanes of paired-end 100 sequencing on the Illumina HiSeq2000 when estimating a yield of 200 million sequences reads per lane. While sequencing cost is constantly decreasing and data output increasing, this is still prohibitive, both in terms of cost and data handling. WGS is therefore unfeasible for efficient detection of off-target effects on a routine basis.

Here, we employed ChIP-seq analysis followed by a targeted sequence capture approach to determine RNA-guided dCas9 specificity and off-target endonuclease activity on a genome-wide scale. We used two replicates for two different gRNA targets, which allowed us to identify gRNA-specific binding as well as binding events that occur independently of the gRNA-specified target sequence. Since neither ChIP-seq binding, nor off-target site prediction based on sequence similarity alone are good predictors for off-target cleavage activity, there is a need for a comprehensive approach to validate a large number of potential off-target sites. We demonstrate that sequence capture can be used as an efficient approach to interrogate Cas9 nuclease off-target activity at a large number of genomic regions simultaneously. This high-throughput approach allowed us to determine and compare cleavage activity at binding sites identified by both ChIP-seq and computationally predicted off-target sites.

## MATERIALS AND METHODS

### Cell culture and transfection

The mouse neuroblastoma cell line Neuro-2a (ATCC #CCL-131) was grown in Dulbecco's modified Eagle's medium (DMEM) supplemented with 10% bovine calf serum (BCS). Neuro-2a cells were grown to 70% confluency and transfected using Lipofectamine 2000 (Life Technologies). HEK293T cells were grown under the same conditions, except DMEM media was supplemented with 10% BCS and 1% penicillin/streptomycin. For ChIP-seq, cells were transfected with 7.5 μg gRNA-expressing plasmid and either 1 or 7.5 μg Flag-tagged dCas9 plasmid per 10-cm dish. Cells were cross-linked 24 or 48 h post transfection (for transfections with 1 or 7.5 μg Flag-tagged dCas9 plasmid, respectively) by incubation with 1% formaldehyde solution for 10 min at room temperature. Cross-linked cell pellets were stored at −80°C.

### Plasmids

Plasmids encoding hCas9-WT and hCas9-D10A were purchased from Addgene (#41815 and #41816, respectively). The double mutant plasmid dCas9 (hCas9-D10A/ H840A) was obtained by Gibson cloning (NEB). Plasmid dCas9-Flag was generated by adding a five amino-acid linker (GGGGS) and 3X Flag tag to the C-terminus of dCas9. The KRAB repressor domain was added upstream of the 3X Flag tag by Gibson cloning resulting in plasmid dCas9-KRAB-Flag. The gRNA cloning vector was purchased from Addgene (#41824) and target-specific gRNA plasmids were created following recommended guidelines (http://www.addgene.org/static/cms/files/hCRISPR_gRNA_Synthesis.pdf). Oligomers containing 20 bp of selected target sequence (e.g. 5′-N_20_ 0f 5′-N_20_-NGG-3′) were cloned into the gRNA cloning vector. The S1 and S2 target sequences are GCTCCCTACGCATGCGTCCC and AATGGCTCAGGTTTGTCGCG, respectively. Oligomers used to create target-specific gRNA vectors are listed in Supplementary Table S1. gRNA plasmid targeting the *VEGFA* site #3 was purchased from Addgene (#47507).

### Single-strand annealing (SSA) recombination reporter assay

The single-strand annealing (SSA) assay is a plasmid-based reporter assay to detect repair of a split luciferase gene as previously described (27). Double strand breaks introduced by targeted Cas9 will allow the SSA repair pathway to reconstruct an active luciferase gene. The SSA reporter plasmid pSSA Rep3–1 is available from Addgene (# 5091). S1 and S2 target sites were introduced between the left and right arms of a split firefly luciferase gene by PCR and cloned into the BglII/EcoRI sites of the vector. HEK293T cells were plated in 24-well plates and co-transfected with 100 ng of gRNA construct, 100 ng of wild-type (WT) Cas9 expressing plasmid, 25 ng of pRL-TKRenilla Luciferase (as a transfection control) and 25 ng of SSA reporter plasmid using Lipofectamine 2000 (Invitrogen). Cells were harvested 48 h post-transfection and lysed in 100 μl of Passive Lysis Buffer (Promega) supplemented with complete protease inhibitors (Roche). Cell lysates (20 μl) were used to determine the luciferase activity using DualGlo reagents (Promega) in a Veritas microplate luminometer (Turner Biosystems). All experiments were performed in duplicates and repeated on at least two different days.

### T7 endonuclease I assay

Neuro-2a cells were co-transfected with plasmids expressing gRNA, WT Cas9 and a green fluorescent protein (GFP; plasmid pCMV-eGFP) using Lipofectamine 2000 (Invitrogen). Cells were harvested 72 h post-transfection and genomic DNA was extracted using the Qiagen Puregene Core kit A according to manufacturer's instructions. The target site region was amplified from 100 ng of genomic DNA (2 min at 95°C; 15 s at 95°C, 30 s at 58°C, 1 min at 68°C, 35 cycles; 5 min at 72°C). Primer sequences are listed in Supplementary Table S1. PCR amplicons were purified using QIAquick PCR Purification kit (Qiagen). A total of 500 ng of purified PCR products were diluted in 1× NEB2 buffer (NEB). The amplicon mixture was heat denatured and slowly reannealed to facilitate heteroduplex formation of WT and mutant alleles (5 min at 95°C; 95 to 85°C at −2°C/sec; 85 to 25°C at −0.1°C/s). The heteroduplex product was digested at the mismatch locus with 10 units of T7 endonuclease I (T7EI) (NEB) for 45 min at 37°C. A control reaction was performed using water instead of T7EI. The digest was resolved by running on a 2% agarose gel, stained with ethidium bromide and visualized using a UV imager. DNA fragments were quantified using the Gel Doc XR imaging system (BioRad) and indel frequency calculated.

### ChIP-seq assay and data analysis

ChIP assays were performed as previously described with minor modifications (28). A total of 50 μg of sonicated chromatin was incubated with 3 μg of monoclonal anti-Flag antibody (SIGMA M2 F1804). After incubation with 3 μg of rabbit anti mouse serum, StaphA cells (Sigma-Aldrich) were used to collect the immunoprecipitates. After washes and reversal of DNA–RNA–protein cross-links, the entire ChIP sample was used to create an Illumina sequencing library using the KAPA library preparation kit (KAPA Biosystems) and NEXTflex DNA barcodes (BIOO Scientific). Quantitative real-time PCR (qPCR) was performed to confirm enrichment of targets in the ChIP libraries as compared to input libraries. Primers to the mouse *Snurf* gene promoter target site were used as positive control primers (Snurf-F 5′-CTCTCCTCTCTGCGCTAGTC-3′ and Snurf-R 5′-AGAGACCCCTGCATTGCG-3′), while a region on mouse chromosome 4 served as a negative control (mmchr4-F 5′-GAGCTATGGCCCATTGATGT-3′ and mmchr4-R 5′-AATAGTGGGATGGTGGGAGA-3′). Libraries were sequenced using the HiSeq 2000 platform (Illumina). Short sequence reads (SR50) were aligned to the mm9 or hg19 genome assembly using bowtie2 (29). Binding sites were identified using MACS1.4 with a chromatin input library as the control dataset (30). Only binding sites that mapped to the non-repeating sequence of the genome were retained for downstream analysis. Overlap analysis was performed using bedtools.

### Data source

The ENCODE ChIP-seq blacklist was obtained at https://sites.google.com/site/anshulkundaje/projects/blacklists (31). DNaseI hypersensitivity data (narrow peak file format) from whole mouse brain were downloaded from the UCSC genome browser hosting the mouse ENCODE project (32). DNaseI hypersensitive sites (DHS) from an adult (week 8) and embryonic (day 14.5) mouse were merged to create a large pool of brain-specific DHS sites. CpG Island coordinates were obtained from the CpGIslandsExt table available at the UCSC Genome browser. All datasets were from the July 2007 mouse assembly (mm9).

### Targeted sequence capture

Neuro-2a cells were co-transfected with equal amounts of plasmids hCas9, gRNA-expression plasmid and pCMV-eGFP. Seventy-two hours after transfection cells were sorted for GFP expressing cells using the Cytomation MoFlo Cell Sorter at the UC Davis Flow Cytometry Shared Resource Laboratory. Genomic DNA was isolated using the Gentra Puregene kit (QIAGEN). Genomic DNA was fragmented to an average size of 150 bp using the BioRuptor NGS (Diagenode). Illumina libraries were prepared with the KAPA library preparation kit (KAPA Biosystems). After ligation to NEXTflex DNA barcodes (BIOO Scientific), DNA was amplified using six PCR cycles following KAPA library preparation kit specifications. Two to four libraries were pooled in equimolar ratios and hybridized to custom designed baits (MYbaits) following the manufacturer's instructions (MYcroarray). Each targeted sequence was 200 bp long and targeted by three 100-bp MYbait oligonucleotides covering the entire length of the targeted region with a 50 bp walking step. Captured libraries were enriched using 15 PCR cycles. As capture efficiency is dependent on experimental conditions, target enrichment was evaluated by quantitative real-time PCR before high-throughput sequencing (Supplementary Figure S8). Enrichment was determined using the 2^∧^-ddCT method comparing amplification of the S1/S2 target region to a promoter region that was not covered by capture baits. We used the same primers as were used for ChIP-seq confirmations. Capture efficiencies were compared for hybridizations at 55, 60 and 65°C. As expected, highest enrichment was observed at 65°C (Supplementary Figure S8). Capture libraries using 60 and 65°C were analyzed by paired-end 100 × 100 and paired-end 150 × 150 sequencing, respectively, using the Illumina 2500 platform.

### Sequence capture data processing and indel analysis

Sequencing reads were split into individual genomic libraries according to index read sequences using publicly available custom python scripts as previously described ((33) and http://comailab.genomecenter.ucdavis.edu/index.php/Barcoded_data_preparation_tools). Briefly, reads were trimmed for quality, the presence of adaptor sequences or N bases and reads that were shorter than 35 bp post-trimming were discarded.

The resulting reads were aligned to the mm9 mouse reference genome using bowtie2 with the *–local* parameter allowing soft clipping of sequence reads. The resulting SAM file, containing information about mapping positions for each read, was screened for the presence of PCR clonal reads as follows: if several read pairs mapped to the same starting positions, and in the same direction, only one of those read pair was retained for downstream analysis. This was performed using a custom python script also available online (overamp.py at http://comailab.genomecenter.ucdavis.edu/index.php/Bwa-doall). The resulting files (non-clonal SAM files) were used for downstream analyses.

Analysis of indel frequencies was performed as follows. First, read pairs for which at least one read mapped at least partially and unambiguously (only one best match found) to the targeted space were selected. Read sequences and associated target peak names were output to a new file. Next, these read-pair sequences were aligned to each other to search for overlap between the two reads. If overlap was found (at least 5-bp overlap), then the two sequences were combined to create a single sequence. If no overlap could be found, the two read pairs were output separately in the same file. Last, the remaining sequences were compared to the reference sequence for each peak to identify polymorphisms and indels. First, the longest region of overlap between the peak and the reference sequence was identified. If the region was <20 bp, the read was not retained for further analysis. Additionally, the region had to at least span 100 bp. For the adequate regions, region sequences were compared between the read and the reference sequences and reads were divided into four categories: reads that matched the reference sequence exactly were labeled as WT, reads that had the same length but differed from the reference sequence by at least one nucleotide were labeled as ‘SNP’ and reads that were shorter or longer than the reference sequence were labeled as carrying a deletion or an insertion, respectively. For each peak, the percentage of indels was calculated by dividing the number of reads labeled as insertion or deletion by the total number of reads containing adequate regions of overlap with the targeted sequences. Targeted sequences containing homopolymers of five or more nucleotides were removed from analysis.

### Targeted amplicon sequencing and indel detection

Genomic DNA was isolated from cells treated with Cas9 nuclease and S1, S2 or empty (control) gRNA as described above. Regions flanking the S1 and S2 target site, as well as the S2 OT1 off-target site were amplified from 200 ng genomic DNA as previously described (27). Primer sequences are listed in Supplementary Table S1. Illumina libraries were prepared and barcoded using the KAPA library preparation kit (KAPA Biosystems) and NEXTflex DNA barcodes (BIOO Scientific). PCR amplification of libraries after adapter ligation was omitted. Libraries were pooled at equimolar concentrations and sequenced using 250-bp paired-end reads on the Illumina MiSeq sequencer. Analysis was performed using a custom bioinformatics pipeline as previously described (27).

### Data access

All ChIP-seq data have been submitted to the Gene Expression Omnibus and are available under accession number GSE61099. Sequence capture data were submitted to the NCBI Short Read Archive (SRA) database under BioProject number PRJNA259773 and SRA ID SRP045878. A website implementing the gRNA selection strategy and Bsite software used to scan the mouse mm9 genome for sequences similar to a given target sequence is available at http://www.genomecenter.ucdavis.edu/segallab/segallabsoftware.

## RESULTS

### Genome-wide analysis reveals Cas9 on- and off-target binding with strongest affinity for the target site

**Figure 1.**
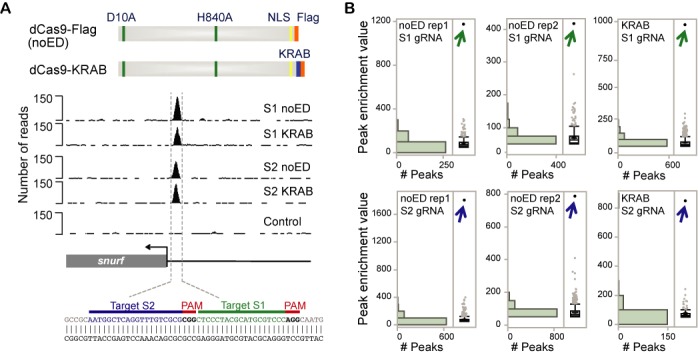
Genome-wide analysis of RNA-guided dCas9 binding in Neuro-2a cells. (**A**) Diagram of the Flag-tagged Cas9 double mutant (dCas9) used in ChIP-seq assay. dCas9 without an effector domain (dCas9-Flag) or fused to a KRAB repressor domain (dCas9-KRAB) are shown at top. Location of nuclear localization domain (NLS) is indicated in yellow, 3X Falg-tag is shown in orange and KRAB domain is high lighted in blue. ChIP-seq profile of dCas9 binding at the on-target site in the mouse *Snurf* locus is shown in the middle. Binding is shown for dCas9-Flag without an effector domain (noED) or with the KRAB repressor domain (KRAB). RNA-guided binding via S1 and S2 gRNAs is depicted. A U6 promoter plasmid that did not express functional gRNA was used as a control. Nucleotide sequence targeted by S1 and S2 gRNA is shown at bottom. (**B**) Distribution of ChIP-seq peak enrichment is shown for two experiments (rep1 and rep2) for dCas9-Flag without an effector domain and for dCas9 fused to the KRAB repressor domain. ChIP-seq enrichment values were obtained using the MACS1.4 peak caller using genomic input DNA as a control. Results are shown for S1 and S2 gRNAs. On-target binding to the *Snurf* promoter is indicated with an arrow.

ChIP assays are most commonly used to detect protein–DNA interactions in cells. In the case of the Cas9:gRNA:DNA complex, we are interested in protein–DNA interactions that are facilitated by the guide RNA. Active Cas9 nuclease was not used in ChIP assays since it induces indel mutations at cleavage sites, which would interfere with Cas9 binding (4,20,21). Therefore, we used a D10A and H840A catalytically inactive dCas9. To create a Flag-tagged dCas9 DNA-binding protein (Figure 1A, top), we fused a short linker (GGGGS) and a 3X Flag tag to the C-terminus of the nuclease-inactive, human codon-optimized Cas9 protein (2). Expression of dCas9 was confirmed by western blot analysis (Supplementary Figure S1A).

Two gRNAs were designed to target two adjacent loci (S1 and S2) within the *Snurf* gene promoter (Figure 1A, bottom, Supplementary Table S1). S1 and S2 gRNAs were carefully selected for their uniqueness in the genome. The closest match to the S1 and S2 target sites in the mouse genome contained three mismatches. To validate that these gRNAs could successfully guide Cas9 to the target site, cleavage activity of WT hCas9 targeted by S1 and S2 gRNA was evaluated in the mouse neuroblastoma cell line Neuro-2a by two methods: (i) the SSA recombination reporter assay and (ii) T7 endonuclease I (T7EI) assay. We found that indel mutations only occurred when Neuro-2a cells were co-transfected with hCas9 and target-specific gRNA, but not when a gRNA control plasmid expressing non-functional gRNA was used (Supplementary Figure S1). After functionality of S1 and S2 gRNAs was confirmed, we performed ChIP-seq assays using the double mutant dCas9 protein (Supplementary Figure S2A). For this purpose, Neuro-2a cells were co-transfected with a plasmid expressing the dCas9-Flag protein and another expressing one of the two gRNAs. ChIP-seq experiments were carried out twice for each gRNA. Experiment 1 was transfected with 1 μg of dCas9-Flag plasmid and harvested after 24 h, while experiment 2 was transfected with 7.5 μg of dCas9-Flag plasmid and harvested after 48 h. The amount of gRNA-expressing plasmid was kept constant at 7.5 μg.

Genome-wide binding sites were identified using the MACS1.4 peak caller using non-enriched chromatin DNA (also referred to as input) as background (30). To reduce the occurrence of false positive peaks, repeat-masked regions were excluded from ChIP-seq analysis (34). To identify unspecific binding regions, ChIP-seq analysis was performed in cells treated with dCas9-Flag and a control U6 promoter plasmid that did not express a functional gRNA (empty gRNA). Only one unspecific peak was identified in this control dataset, which was removed from all other datasets. The number of ChIP-seq reads and the number of ChIP-bound regions are summarized in Supplementary Table S2. ChIP-seq analysis of dCas9-Flag binding guided by S1 gRNA resulted in 338 and 517 peaks, while S2 gRNA guided binding resulted in 737 and 1009 peaks (experiments 1 and 2, respectively). The presence of more dCas9-Flag protein in experiment 2 correlates with an increase in ChIP-seq binding sites.

For both gRNAs, the top-ranked binding site in each dataset was an identical match to the target locus, demonstrating high affinity for the on-target site (Figure 1A, middle and Figure 1B). In experiment 1, for which lower amounts of dCas9-Flag had been used, the target site was enriched four and six-fold higher than the highest off-target binding site for S1 and S2, respectively. The target site enrichment was only two-fold higher than the highest off-target site in experiment 2, consistent with the idea that reducing protein and/or gRNA amounts and exposure time can limit off-target binding (5,18,19,22).

### ChIP-seq identifies gRNA-specific and gRNA-independent binding sites

We also wanted to explore the possibility that some of the off-target binding sites represented dCas9 binding events that were not specific to a given gRNA or target sequence. To identify possible sites of gRNA-independent recognition, we searched for peaks that occurred in both the S1 gRNA and S2 gRNA datasets and identified 150 binding sites that were common for both gRNAs (Supplementary Figure S2A). These gRNA-independent peaks were subtracted from the S1 and S2 datasets and were analyzed separately (Supplementary Table S3). Consequently, 274 and 404 peaks were targeted only by S1 gRNA, and 665 and 883 peaks were targeted only by S2 gRNA (experiment 1 and 2, respectively; peak numbers are summarized in Supplementary Table S2). We next overlapped gRNA-specific peaks from the two experiments to obtain 69 S1-specific and 254 S2-specific *bona fide* binding sites (Supplementary Figure S3). These overlaps correspond to 25 and 38% for S1 and S2 datasets, respectively and represent a robust set of peaks for more in-depth analysis. We used input as background for peak calling to minimize the occurrence of false peaks. The observation of only one unspecific peak in the control ChIP-seq dataset suggests that binding sites that were only observed in one dataset (non-overlapping peaks) may be due to transient interactions between CRISPR/Cas9 and genomic PAM sites or regions with partial matches to the target site adjacent to a PAM site. We focused all downstream analysis on gRNA-specific binding sites that occurred in two independent ChIP-seq experiments (69 and 254 binding sites for S1 and S2, respectively).

**Figure 2.**
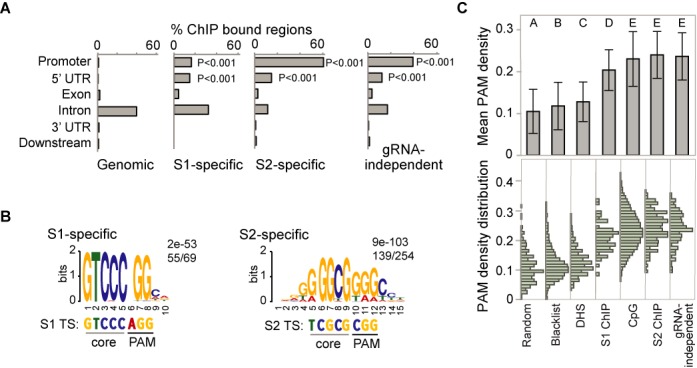
Identification of G- and C-rich motifs at off-target binding sites correlates with increased PAM motif density. (**A**) Location analysis of ChIP-seq binding sites. ChIP peaks at gene-proximal regions and within gene bodies were analyzed using Cis-regulatory element annotation system (CEAS). Gene-proximal regions include promoters up to 1 kb upstream of the transcription start site and regions up to 1 kb downstream of the transcription termination site. Binding within gene bodies is divided into four functional categories: 5′UTRs, exons, introns and 3′UTRs. Localization of ChIP regions within these categories is compared with the genome background percentages for the same categories. *P*-values for the significance of the relative enrichment with respect to the background were calculated using one-sided binomial test and are indicated for categories with significant enrichment in ChIP samples as compared to genomic background. The remaining ChIP bound regions do not fall into the above categories and are considered ‘distal intergenic’ regions. No significant enrichment was observed at distal regions as compared to background. (**B**) Identification of *de novo* motifs for overlapping dCas9 binding sites in Neuro-2a cells. MEME-ChIP motif analysis using the central 100 bp of S1 and S2 gRNA-specific ChIP-seq binding regions reveals the indicated motif. The statistical significance of the motif is indicated by *E*-values, as output by MEME-ChIP. The number of sequences containing the identified motif relative to the total number of binding sites is indicated. The 5-bp core sequences and PAM for the target sites (TS) are shown. (**C**) PAM density plot based on occurrence of NGG in 100-bp regions in the genome. Both DNA strands were scanned for the three-nucleotide motif. PAM density of the central 100 bp of gRNA-specific and gRNA-independent ChIP-seq peaks was determined for S1-specific (*n* = 69), S2-specific (*n* = 254) and gRNA-independent (*n* = 150) binding sites, Randomly selected 100-bp regions of the mouse genome (*n* = 340) and an array (*n* = 3038) of artefact peaks (also known as ENCODE ChIP-seq blacklist) were used as a control. PAM density was also calculated for CpG islands (*n* = 16026) and DNase I hypersensitive sites (DHS, *n* = 469 449). Mean of PAM density +/− standard deviation is shown in the top panel and statistical groupings A-E as determined by student's *t*-test are indicated above each column. Different letters indicate statistically different means between samples. PAM density distribution is plotted in the lower panel.

To determine functional genomic regions bound by Cas9, we used the cis-regulatory element annotation system (35,36). Gene-proximal regions include gene promoters up to 1 kb upstream of the transcription start site, gene bodies and downstream regions up to 1 kb downstream of the transcription termination site (Figure 2A, Supplementary Table S3). The majority of gRNA-specific as well as gRNA-independent ChIP peaks localize to gene-proximal regions with 70, 92 and 74% of S1-specific, S2-specific and gRNA-independent binding sites, respectively. Functional regions of gene bodies include 5′ UTRs, coding exons, introns and 3′UTRs. Localization of ChIP regions within these categories is compared with the genome background percentages for the same categories and *P*-values are calculated. ChIP peaks show significant enrichment at proximal promoters and in 5′UTRs, while <5% of exons and <1% of 3′UTRs were targeted (Figure 2A). Binding to intronic regions was not significantly enriched when compared to genomic background.

### The KRAB effector domain does not increase dCas9:gRNA recruitment to DNA

The nuclease-inactive dCas9 can be used as a transcriptional regulator when fused to an effector domain, which can either up or down regulate transcription (7–9) or alter the epigenetic landscape surrounding the target site. Our study was carried out with dCas9-Flag that does not contain an effector domain (noED) to identify direct interactions with genomic DNA. But it has been of concern that effector domains themselves could contribute to the recruitment of fusion proteins to DNA resulting in additional set of off-target binding sites specific to the effector domain. Increased binding to the genome has been observed when an artificial zinc finger protein targeting the human Sox2 promoter was fused to the super KRAB domain (37). To test the hypothesis that the KRAB domain could increase dCas9 binding, we used the dCas9 protein fused to the KRAB repressor domain and 3X Flag tag (Figure 1A, top; Supplementary Figure S4). Expression of dCas9-KRAB was confirmed by western blot analysis (Supplementary Figure S1A). ChIP-seq experiments were conducted using dCas9-KRAB with either S1 or S2 guide RNAs. After removal of previously identified gRNA-independent peaks, 617 and 135 binding sites were identified for S1 and S2 gRNA, respectively. Genome-wide binding profiles using the dCas9-KRAB fusion protein showed the same distribution as the ones that did not contain an effector domain. The on-target site was by far the most highly enriched genomic region and was at least three-fold higher than any off-target binding site (Figure 1A, middle and Figure 1B). We then compared ChIP peaks bound by dCas9-KRAB to the high-confidence ChIP peaks bound by dCas9 without the effector domain. When comparing S1 peaks, 83% of 69 S1-specific binding sites were also occupied by dCas9-KRAB. For S2-specific binding, 57 of the 135 regions bound by dCas9-KRAB were previously identified high-confidence S2 peaks. The lack of highly enriched ChIP peaks in addition to the target site indicates that the KRAB effector domain was not responsible for detectable recruitment of dCas9 to genomic loci. Future studies will elucidate if this holds true for other effector domains such as activators and epigenetic modulators.

### ChIP-seq identifies a 5-bp core seed region for RNA guided Cas9 binding

The binding profiles for both gRNAs revealed that enrichment values for the target site were by far the highest signal genome-wide (Figure 1B). The lower enriched off-target binding sites may reflect transient interactions between dCas9 and the genome or may be stable interactions only occurring in a few cells at a time. To identify common features of ChIP binding sites, we performed *de novo* motif analysis on the ChIP peaks identified. We retrieved 100-bp sequences centered around the peak middle of Cas9-bound regions and identified the most significant motif using MEME-ChIP (38) (Figure 2B). Out of 69, 55 S1 binding sites contained a motif of GTCCCHGGCD (*E*-value 2.1e-53). Interestingly, this motif is identical to the 5-bp sequence immediately upstream of the PAM site (NGG) present in the target site sequence CTCCCTACGCATGCGTCCC(AGG) (Figure 1A, bottom). Most studies identified a longer seed region between 10 and 12 bp (5,14,16). We therefore defined the 5-bp directly adjacent to the PAM as the core region. Our observation of a 5-bp core region is consistent with the 5-bp ‘seed’ motif reported in another ChIP-seq study (22). Motif analysis of the S2-bound regions identified the motif BRRKGGGCGGRGCYD in 139 of 254 binding sites (*E*-value 8.9e-103). There is no obvious resemblance between the identified motif and the 5-bp core region of the S2 target site sequence AATGGCTCAGGTTTGTCGCG(CGG). Moreover, it is almost identical to the motif identified in 70 of 150 gRNA-independent binding sites: GGGCGKRGMYD (*E*-value 2.6e-16) (Supplementary Figure S5). When using the Tomtom Motif Comparison Tool (39) the motif identified in S2-specific and gRNA-independent binding sites resembles transcription factor motifs, such as SP1 and KLF5 motifs (Supplementary Figure S5). SP1 motifs are primarily found in promoter regions, where SP1 interacts with the basal transcriptional machinery (40). Since Cas9 binding was primarily localized to promoter regions, promoter-specific motifs such as an SP1 binding site may skew the results of *de novo* motif analysis.

**Figure 3.**
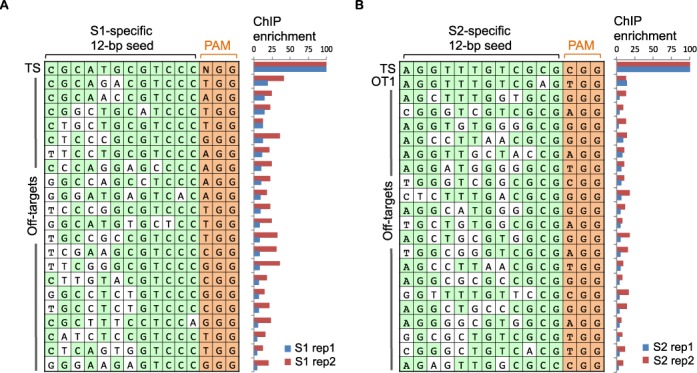
Genomic sequences bound by Cas9:gRNA. Comprehensive list of sequences in ChIP-bound regions that contained a match to the 12-bp seed sequence of the target site when allowing up to four mismatches to S1 (**A**) and S2 (**B**) target sites. Nucleotides identical to the target site are highlighted in green. The PAM (NGG) is depicted in orange. Bar graphs on the right display percent ChIP-seq enrichment relative to the target site (TS, top bar). Enrichments are shown for two independent experiments, rep1 and rep 2. Rep1 was obtained after exposure to lower amounts of dCas9 expressing plasmid (1 μg) for 24 h, while rep2 was transfected for 48 h with 7.5 μg dCas9 expressing plasmid.

We therefore adjusted our analysis of ChIP-bound regions by specifically searching Cas9-bound regions for the presence of partial matches to the target site. It has been shown that the seed region, i.e. the region directly upstream of the PAM site, is most important for target site recognition. We first looked for sequence similarity allowing up to four mismatches to the 12-bp seed region in S1 ChIP-seq peaks, more specifically in 100-bp sequences centered on the middle of ChIP-seq peaks. Among 68 S1 off-target binding sites, 21 sites (31%) contained a 12-bp sequence with up to four mismatches; two sites had two mismatches, four sites had three mismatches and the rest had four mismatches (Figure 3; Supplementary Table S4). When Cas9-bound sites were interrogated for presence of the 5-bp core region adjacent to a PAM, 51 (75%) of the 68 S1 off-target regions contained a perfect match to GTCCC(NGG). A second PAM with the three-nucleotide sequence NAG has been reported as an alternative to NGG (14). When searching the 68 S1 off-target regions for a perfect match to the sequence GTCCC(NAG), we identified only two sites, suggesting that the PAM motif NAG does not play a major role in target recognition.

We then performed the same analysis for S2 gRNA-specific ChIP bound regions. When allowing four mismatches, only 21 of the 253 off-target sites contained a sequence similar to the 12-bp seed region adjacent to the PAM motif (NGG) (Figure 3; Supplementary Table S4). On the other hand, 12 binding sites matched perfectly to the 5-bp core region adjacent to the PAM motif, TCGCG(NGG) and another 106 sites exhibited only one mismatch to the core region. The fact that only few binding sites match the 5-bp core region adjacent to a PAM and that the motif for S2 and gRNA-independent binding sites are identical suggests that a subset of S2-specific binding sites might be gRNA-independent binding sites, which we were unable to identify.

To demonstrate the reproducibility of genome-wide dCas9 binding analysis in a different cell line using a different gRNA, we performed ChIP-seq analysis using *VEGFA* #3 gRNA (16) in the human HEK293T cell line. As before, we observed clear binding to the target site and significantly lower enrichment at off-target binding sites (Supplementary Figure S6A). After overlapping two biological replicates, we obtained 16 high confidence peaks. MEME-ChIP analysis identified TSCGTGKGGSSGGRR as the predominant motif in all 16 binding sites, which contains the 6-bp core motif of the *VEGFA* #3 target site GGTGAGTGAGTGTGTGCGTG(TGG) (Supplementary Figure S6B). Interestingly, the motif extends beyond the 3′ end of the PAM site. The *de novo* motif identifies a GGGG stretch starting three bases downstream of the PAM further suggesting the importance of G-rich sequences for Cas9 target recognition.

### The PAM NGG is over-represented in Cas9:gRNA bound regions

To investigate the correlation between G- and C-rich regions bound by Cas9:gRNA and the occurrence of NGG PAM sites, we calculated PAM site density by counting the frequency of PAM sites (NGG) in 100-bp sequences centered on the middle of each ChIP-seq peak, on either DNA strand. The median PAM densities were higher (20% at S1-specific peaks and 24% for S2-specific and gRNA-independent peaks) in off-target binding regions compared to random genomic regions (median of 9%) (Figure 2C). We also compared our binding sites to three other sets of sequences: (i) Blacklist regions of the mouse genome were previously identified as signal artefacts in next generation sequencing experiments, independent of cell line and experiment (https://sites.google.com/site/anshulkundaje/projects/blacklists). There was no overlap between ChIP-seq peaks in our study and blacklist regions identified as part of the ENCODE project (31) and the density of PAM sites in this list of sites was 11%. (ii) Recently, a correlation between regions of DNaseI hypersensitivity and off-target binding was reported (22). Because there are no DHS data available for the Neuro-2a cell line used in this study, DHS from two brain datasets (adult and embryonic) were combined instead. In those, PAM motif density was 12%. (iii) CpG islands had a PAM density of 24%. Student's *t*-tests (*P* < 0.0001) were conducted to compare PAM densities between different sequence datasets. There was no significant difference in PAM density between CpG islands, S2-specific peaks and gRNA-independent peaks (Figure 2C). All peak sets and CpG islands were significantly enriched in PAM sites compared to the other three categories (the ENCODE blacklist, the DHS and the random set).

### Off-target binding correlates with accessible chromatin and GC-skewed genomic regions

Chromatin accessibility as assayed by DNaseI hypersensitivity had been found to be a strong indicator for *in vivo* Cas9 binding (21,22). We therefore overlapped off-target binding sites identified by ChIP-seq with DHS from mouse brain available from ENCODE (32). Indeed 85% of S1, 96% of S2 and 92% of g-RNA independent off-target binding sites localize to accessible chromatin regions, confirming a strong positive correlation between accessible chromatin and off-target binding.

Our study and the work of others suggest that chromatin accessibility plays a critical role in Cas9 binding to off-target sites. While Cas9 cleavage activity was reported at methylated target sites (41), a genome-wide study of Cas9 binding has reported a negative correlation between off-target binding and DNA methylation (22). It has been proposed that Cas9 binding to the PAM site triggers local unwinding of DNA, which allows the gRNA molecule to pair with one complementary strand of double stranded DNA, forming an R-loop (4,5,42). In mammalian genomes, R-loop formation has been implicated in the protection of CpG island promoters (CGI) from DNA methylation (43). Regions with GC skew displaying strand symmetry in the distribution of G and C nucleotides are an attribute of human unmethylated CGI and are used as a predictive feature of R-loop formation (43,44).

GC skew is a DNA sequence characteristic measuring the strand bias in the distribution of G and C residues. To investigate if there was a correlation between off-target binding sites identified by ChIP-seq and genomic regions with GC skew, regions of GC skew were identified in the mouse genome using the SkewR algorithm as previously described (43). 43, 89 and 70% of S1, S2 and gRNA-independent off-target binding sites localized to GC skew regions, respectively. Since GC skew is predictive of co-transcriptional R-loop formation (43,44), these observations suggest that preferential off-target binding may occur at regions already occupied by RNA:DNA hybrids. GC-skewed R-loop-prone regions are highly enriched in CpG islands, 5′-UTRs and DNaseI accessible regions which are features associated with off-target binding sites in this study (Figure 2) and by another group (22). Interestingly, the motifs identified as highly enriched in the S2-specific and gRNA-independent datasets (Figure 2A, Supplementary Figure S5) were highly GC-skewed and each carried two consecutive clusters of at least three guanines, making these motifs ideal candidates for initiating R-loop formation (45). At the same time, GC-rich, GC-skewed regions such as CpG islands show elevated PAM motif density (Figure 2C), making them ideal off-target decoys from a DNA sequence, structural (R-loop) and chromatin viewpoint.

### Cas9-bound regions and sequence-based predicted off-target sites correlate poorly

If sequence similarity to the target site is the main determining factor for off-target binding, one would expect these sites to be bound by Cas9:gRNA in a ChIP-seq experiment. To identify genomic regions that are similar to the target site sequence, we scanned the mouse genome for genomic loci with up to four mismatches to the target site adjacent to a PAM. Since the target regions were carefully chosen for their uniqueness in the genome, 117 and 28 sequences were identified with three or four mismatches to the target sites S1 and S2, respectively. There were no sequences identified with less than three mismatches to the target sites. For a more comprehensive comparison between predicted off-target sites and ChIP-bound regions, we used all ChIP-seq peaks from both experiments for each gRNA. Only one predicted off-target site was present in the S2 ChIP-seq dataset and none in the union of S1 peaks. Interestingly, the one site that was in common between S2-specific ChIP-bound regions and computationally predicted sites was the same off-target site that contained the best match to the 12-bp seed region (Figure 3B, Supplementary Table S4). We labeled this common off-target site as OT1. OT1 was bound with about eight-fold lower affinity in ChIP experiments as compared to the S2 target site. In fact, based on peak enrichment values OT1 was ranked number 10 in experiment 1 and number 140 in experiment 2 for ChIP enrichment in the S2 gRNA specific dataset. The low enrichment values suggest that mismatches destabilize the Cas9:gRNA:DNA interaction.

### Sequence capture as an efficient method to identify indels at potential Cas9 off-target sites

Next, we wanted to evaluate sequence capture as a high-throughput approach to simultaneously screen hundreds of potential off-target regions for indels and assess Cas9 activity at potential off-target sites (46). While exome capture has been used to screen for Cas9 induced indels in transcribed regions of the genome (15), custom capture allows us to interrogate very specific loci. Location analysis of ChIP-seq binding sites showed that only 4.4, 2.8 and 3.4% of binding sites (S1, S2 and gRNA-independent sites, respectively) are found in coding exons (Figure 2A). Targeted sequence capture allowed us to screen off-target binding sites identified by ChIP-seq as well as computationally predicted off-target sites. Capture baits were designed to cover 200-bp regions centered on each potential off-target site. Baits were 100 bp in length overlapping by 50 bp. Three baits were designed to cover each 200-bp genomic region.

Custom capture baits were designed to survey 1200 genomic loci including 473 sites identified by ChIP-seq (69 for S1, 254 for S2, 150 for gRNA-independent peaks), 310 computationally predicted sites (170 for S1, 140 for S2) and 430 random control regions. Illumina genomic sequencing libraries were prepared from two independent capture experiments. Each experiment consisted of four samples including untreated Neuro-2a cells, Neuro-2a cells expressing Cas9 endonuclease together with either empty, S1 or S2 gRNA. Capture libraries were pooled and analyzed by high-throughput sequencing on the Illumina HiSeq 2500 platform. Sequence reads from both experiments were combined, filtered, trimmed based on read quality and aligned to the mm9 mouse genome (UCSC).

In the case of PCR-based assays, identical amplicons are generated, making it impossible to identify clonal reads, i.e. products of PCR amplification of the same initial molecule. High numbers of sequences can be analyzed but this can artificially affect the results in a random manner. On the other hand, reads obtained from sequencing libraries are generated through random shearing of DNA and are therefore staggered. Identical reads generated by amplification during library preparation can therefore be identified by the fact that they span the exact same DNA sequence. Clonal reads were removed from all of our datasets. Removing clonal reads is crucial to the analysis of indel frequencies and it is important to note that results obtained from sequence capture cannot be directly compared to results obtained by the targeted PCR-based sequencing assay.

**Figure 4.**
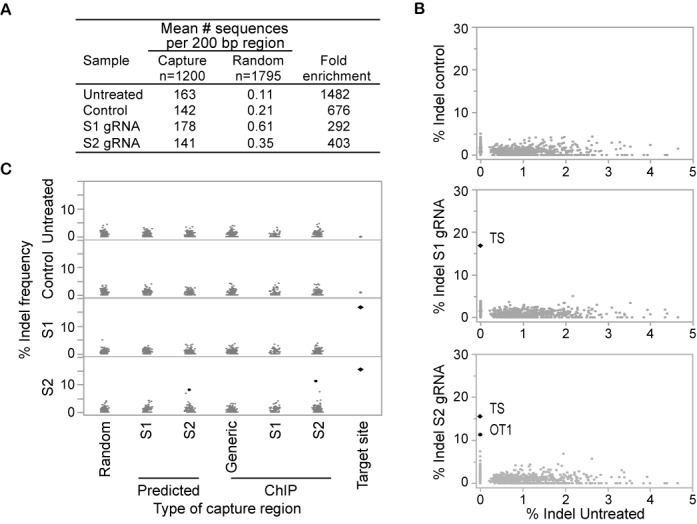
Sequence capture identifies indels at the target sites and one off-target site. (**A**) Sequence coverage of capture regions. The mean number of sequence reads mapping to each 200 bp target region (*n* = 1200) is shown for untreated Neuro-2a cells, cells treated with catalytically active Cas9 and non-functional empty gRNA, S1 gRNA or S2 gRNA. As a comparison, coverage was also calculated for a random set of non-targeted genomic regions of the same size (*n* = 1795). Fold enrichment of capture regions as compared to random regions is shown. (**B**) Bivariate analysis of percent indels relative to the total number of reads per target region. The top panel depicts a comparison of Neuro-2a control cells expressing catalytically active Cas9 and non-functional gRNA to untreated Neuro-2a. The middle panel shows a comparison of cells treated with S1 gRNA against untreated Neuro-2a cells. High occurrence of indels is observed for the target site (TS). The bottom panel shows a comparison of cells treated with S2 gRNA to untreated Neuro-2a cells. High indel frequencies are observed for one off-target site (OT1) in addition to the TS. Indels at the S1 and S2 target sites and the OT1 S2 off-target site are statistically significant as determined by Fisher's exact test (*n* = 1200, *p* < 0.01). (**C**) Occurrence of indels at computationally predicted off-target sites and Cas9-bound sites identified by ChIP-seq is compared to indel frequency at the target site and at random genomic regions. Indel frequencies for each category are compared across the four treatments: untreated Neuro-2a cells, control Neuro-2a cells treated with Cas9 and empty gRNA (top two panels) or with Cas9 and S1 gRNA or S2 gRNA (bottom two panels). Indels with statistical significance as determined by Fisher's exact test (*n* = 1200, *P* < 0.01) are indicated as black dots.

To assess the efficiency of the capture reaction, coverage of targeted regions relative to randomly chosen regions was calculated. The mean number of reads mapping to each 200-bp target region ranged from 141 to 163 reads, while randomly chosen genomic regions exhibited a mean read count of less than one (Figure 4A), which corresponds to an enrichment of captured regions ranging from 292 to 1482-fold.

To identify indels, we first selected read pairs for which at least one of the two reads unambiguously mapped one of the captured regions. Of these sequences, we identified read pairs for which the forward and reverse reads overlapped, in which case they were joined to generate one long sequence. The non-overlapping pairs were kept as separate reads. These sequences were then compared to the target sequence. Reads that were identical matches to the target sequence were identified as WT. Sequences containing single nucleotide polymorphisms (SNPs) as compared to the reference were recorded, but were not considered a result of Cas9 cleavage activity. Insertions and deletions were identified and the percentage of indels was calculated relative to all reads matching each target region. A total of 44 of the 1200 capture regions showed indel frequency >5% with indel frequencies as high as 80%. This is most likely due to mismatches between the Neuro-2a cell line and the mouse reference genome or spontaneous mutations arising in cell culture. These regions have thus been omitted from analysis. A minimal threshold of 25 sequence reads per target region was applied for indel analysis. A certain level of background indels has been reported previously (15,18,22,47) and is mostly thought to be the result of sequencing or indel calling errors, while other indels simply represent differences between the sequenced and the reference genome. Furthermore, it has also been reported that indel rates can increase to about 2% in homopolymer stretches (47). Since we observed an elevated indel frequency next to homopolymers (defined here as a stretch of at least six identical consecutive bases) in all samples including untreated cells, we omitted these targeted sequences from analysis. After these filtering steps, our capture analysis focused on 910 genomic regions. We then compared indel frequency in gRNA-treated samples with indels in untreated samples and applied the Fisher's exact test to identify genomic regions that had statistically significant enrichment in indels (*n* = 1200, *P* < 0.01).

**Figure 5.**
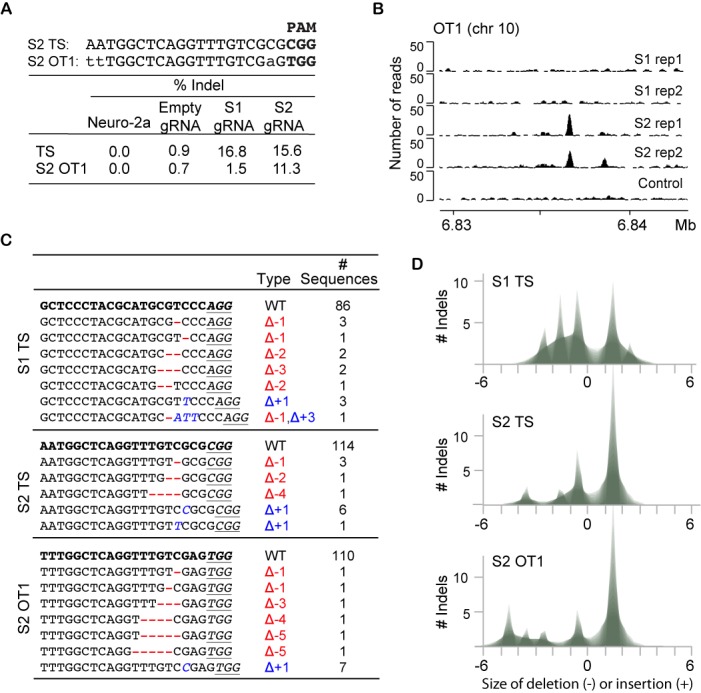
Sequence capture identifies indels at the target sites and one off-target site. (**A**) A comparison of S2 TS and OT1 sequences, and a table listing the percentage of indels calculated for each of the four datasets at S1 and S2 TS and S2 OT1. (**B**) Browser image depicting the position and height of ChIP-seq peaks at the off-target site OT1 in the mouse genome (mm9 assembly). Binding is specific to cells treated with S2 gRNA and is not present in control cells or cells treated with S1 gRNA. (**C**) Table summarizing indels identified by targeted sequence capture at the S1 and S2 target sites (TS) and S2 off-target site (S2 OT1). The number of sequences is listed for WT sequences and sequences with indels. Deletions are indicated by dashes and insertions are shown in italic letters. One sequence carries both an insertion and a deletion. The three-nucleotide PAM sequence is underlined where present. (**D**) Shadowgram depicting indel size distribution at the S1 and S2 target sites as well as the S2 off-target site OT1. Indel sizes are indicated on the x-axis. Deletions are given as negative values, while insertions are positive values. The number of indels is plotted on the y-axis.

In Neuro-2a cells treated with S1 or S2 gRNAs, the two on-target sites were identified as statistically significant with an indel frequency of 16.8 and 15.6%, respectively (Figure 4B and C). While no significant off-target cleavage was observed for S1 gRNA, one off-target site was identified for S2 gRNA. In this experiment, the indel frequency of 11.3% observed at the off-target site (OT1) was only slightly lower than 15.6% at the target site (Figures 4B and 5A). No indels with statistical significance were identified when the reverse analysis was carried out, i.e. when limiting the analysis to samples with <5% indel frequency in the treated samples and searching for sequences with significantly higher frequencies of indels in the untreated sample (Supplementary Figure S7). In addition, no significant difference was observed when comparing indel frequencies of untreated Neuro-2a cells to control cells treated with Cas9 and empty gRNA. We then took a closer look at indel frequency dependent on the type of capture region (Figure 4C). As expected, significant indel enrichment occurred at the target sites and at the S2 off-target site OT1. Indels at OT1 were identified in both categories, S2 ChIP-seq and computational prediction. The indel occurrence varies slightly with 11.3% for Cas9 bound regions and 8.1% for computational predicted off-target sites (Figure 4C). This difference is due to a 29-bp shift in chromosomal coordinates based on the two different approaches used to identify these regions. Indel analysis by high-throughput sequencing of amplicons fully confirmed OT1 as a *bona fide* S2 specific off-target site (Supplementary Figure S9). The indel frequency was 34 and 35% at the S1 and S2 target site, respectively, while the off-target site OT1 showed indels at a frequency of 16% in S2 treated cells and was undetectable in S1 treated cells.

The OT1 off-target site that was identified by ChIP-seq and validated by sequence capture shows three mismatches to the target sequence. Two mismatches occurred at the very 5′ end, furthest away from the PAM site, while the third mismatch occurred in the 5-bp core region (Figure 5A). It has been reported that off-target activity is more likely to occur when mismatches between the guide RNA and the template are not adjacent to the PAM site, but rather in the PAM distal region (14,16,18). However, OT1 also contains a mismatch just 2 bp from the PAM site. OT1 is specific to S2 gRNA in ChIP-seq as well as Cas9 activity assay. No ChIP binding to OT1 was observed using S1 gRNA or control gRNA (Figure 5B).

It is important to note that results obtained from sequence capture cannot be directly compared to results obtained by the targeted PCR-based sequencing assay. The background indel frequency in our sequence capture experiments varied from 1 to 5%, while the indel frequency in the PCR-based assay is reported as 0.1%. One of the major advantages of sequence capture compared to amplicon sequencing is the ability to computationally screen sequencing data for the presence of clonal reads. Clonal reads are produced by PCR amplification of the same original fragments, which can introduce a significant bias in indel frequency, independently of the actual frequency present in the sample. We wanted to investigate if the presence of clonal reads influences indel frequency. To assess the effect of clonal reads, we performed indel analysis on a set of capture data before (Supplementary Figure S10A, left panel) and after removal of clonal reads (Supplementary Figure S10A, right panel). While the level of noise was visibly reduced when analyzing clonal reads (Supplementary Figure S10B), indel analysis resulted in detection of indels at the same sites, the expected target sites and one additional off-target site (S2 OT1).

### Cas9-induced indels localize to central positions in captured regions

The majority of indels identified by sequence capture were small deletions ranging from 1 to 5 nt (Figure 5C and D). We also observed small insertions of 1 nt as well as combinations of small insertions and deletions. Since sequence capture relies on hybridization between capture baits and complementary genomic sequences, this method preferentially enriches for sequences with only small mismatches to the capture region. RNA-guided Cas9 frequently introduces small indels ranging from 1 to 5 bp, while larger indels are observed at much smaller frequencies (17,48). Sequence capture efficiently identifies small Cas9-induced indels, but may be limited in the identification of larger insertions and deletions.

**Figure 6.**
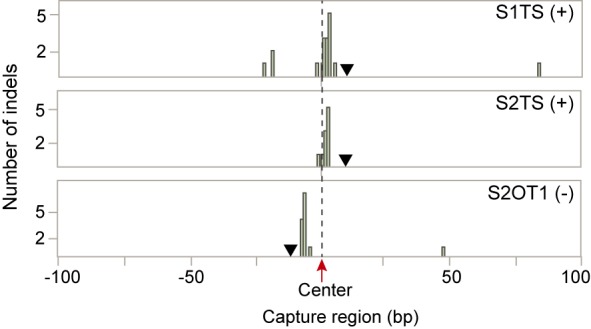
Cas9-induced indels occur proximal to the center of 200-bp capture region. The number of identified indels is plotted relative to the center of 200-bp capture region. Cas9-induced indels observed at the target sites and the S2 off-target site OT1 localize in proximity to the capture region center. (+) and (−) describe on which DNA strand the NGG PAM is located. The PAM site is indicated by a triangle.

Since capture regions were centered on either the ChIP-bound site or the computationally predicted sequence, the Cas9 induced indel is expected to occur close to the center of the 200-bp regions. Indels identified on the S1 and S2 target sites and S2 off-target site (OT1) were indeed close to the peak center with the majority just a few base pairs away (Figure 6). The S1 and S2 target sites are on the sense strand, while the S2 OT1 is on the antisense strand. Therefore, indels are observed a few base pairs upstream of the center of the OT1 capture region while they cluster a few base pairs downstream of the center of the S1 and S2 target sites. This is consistent with the cleavage profile of Cas9, which has been shown to cleave three bases upstream of the PAM site.

Even though we found poor correlation between ChIP-bound regions and predicted off-target sites overall, the one site that was identified by both methods (OT1) has been confirmed by the Cas9 cleavage assay. Our data suggest that off-target site prediction based on sequence similarity to the target site in combination with sequence capture can be used as an efficient approach to determine Cas9 specificity. Future work will explore if this holds true for a larger selection of gRNAs.

## DISCUSSION

We have demonstrated that genome-wide ChIP-seq analysis as well as computational off-target prediction can be used in combination with targeted sequence capture to assess Cas9 cleavage activity at a large number of potential off-target sites. Custom sequence capture offers several advantages over other methods determining Cas9 specificity. There are several low throughput methods available that interrogate one locus at the time, such as the T7 endonuclease I assay or amplification of the target region by PCR followed by sequencing. On the other hand, WGS can be used to identify indels in an unbiased manner, but is costly and lacks sequencing depth. For these reasons, targeted sequence capture offers an attractive alternative method to enrich and efficiently interrogate hundreds or thousands of genomic loci simultaneously. In our capture experiments, target site enrichment was 300-fold or higher. While WGS requires ∼4.5 lanes of paired end 100 × 100 sequencing for one sample, the enrichment of 1200 genomic loci during sequence capture allows us to achieve 150× coverage or higher in only a fraction of a sequencing lane. Custom capture designs are available ranging from 20 000 to 200 000 custom baits, which translates to screening for indels of ∼6670–66 700 genomic loci. This could be a very powerful approach to survey off-target activity for a multitude of gRNAs and test Cas9 and/or gRNA variations to help improve target specificity.

Previous studies showed that ChIP enrichment and cleavage activity are not necessarily correlated (20–22,40). It was therefore important to screen indel frequencies at 473 loci identified by ChIP-seq and 310 off-target sites predicted by similarity to target sequence. We confirmed Cas9 cleavage activity at the target sites of both gRNAs and identified a single off-target site (OT1). This off-target site was also the only site that was identified using both the genome-wide ChIP-seq binding analysis and the sequence-based *in silico* prediction. Additionally, we found that binding of Cas9 protein does not necessarily translate into Cas9 nuclease activity. The high enrichment of the target site sets it apart from all off-target sites. This suggests that stable interactions only occur between Cas9:gRNA and the exact target site. Off-target sites that differ from the target site by three or more mismatches do not form as stable interactions, which is reflected in much lower ChIP enrichments and the lack of detectable cleavage activity. OT1 was enriched at much lower levels than the target site in ChIP experiments but cleavage still occurred at high frequency. However, the current analysis did not consider chromatin conditions or local DNA topology, which may have influenced cleavage activity at this site. Currently, introduction of indels by Cas9 is used to determine off-target activity. However, the question remains whether dCas9 fusion proteins with e.g. epigenetic modulators display similar binding specificity or perhaps have increased off-target activity.

We have identified target and off-target indel frequencies induced by Cas9 that were statistically higher than in untreated or control cells. The sequence capture approach used in this study shows a background frequency of indels similar to those previously reported when exome capture or WGS were used to identify Cas9 induced indels (15,24–26). Cas9 induced indels present a distinct signature since Cas9 causes a double strand break 3-bp upstream of a PAM and within regions with at least limited similarity to the target site. Indels were frequently observed at positions other than those expected from Cas9 mediated double strand breaks, confirming that they are probably the result of sequencing errors or correspond to mismatches between the sequence of the strain at hand and the reference genome sequence (24,49,50). These elements contribute to the background of indels observed in untreated cells as well. Further optimization of the experimental conditions and/or refinement of the indel analysis pipeline will help lower the occurrence of background indels. The current design of capture probes focused on 200-bp regions centered on the target and potential off-target sites. Targeted sequence capture can identify chromosomal rearrangements when probes are designed with much tighter spacing of 1–3 bp spacing (51). The probe spacing in our capture design was 50 bp. Using a much closer spacing strategy will most likely identify larger insertions and deletions as well as detect rearrangement breakpoints.

A great deal of future research will focus on improving and predicting Cas9 specificity for a given gRNA as Cas9 holds potential for developing gene and cell therapy. While there is a lot of evidence supporting the high specificity of Cas9, there are a handful of examples revealing high off-target activity. It is still unclear why RNA-guided targeting results in high off-target activity for one gRNA, but not for another. It is certainly important to carefully design gRNAs based on the uniqueness of its target sequence in the genome. Careful gRNA design can be combined with other off-target limiting methods. For example, it has been shown that by simply using a shorter complementary gRNA of 18 nt instead of 20 nt, off-target effects became undetectable while on-target activity remained the same (52). Another study also reported that shorter gRNA sequences reduced off-target effects, but found that on-target activity was reduced as well (18). On the other hand, addition of two G nucleotides at the 5′ end of the gRNA sequence was found to increase target site specificity (15). As an alternative to Cas9 nucleases that introduce double strand breaks, the use of paired Cas9 nickases significantly reduced off-target activity (15,53). However, our results demonstrate that even a simple configuration of a Cas9:gRNA nuclease can support very specific DNA cleavage activity.

**Figure 7.**
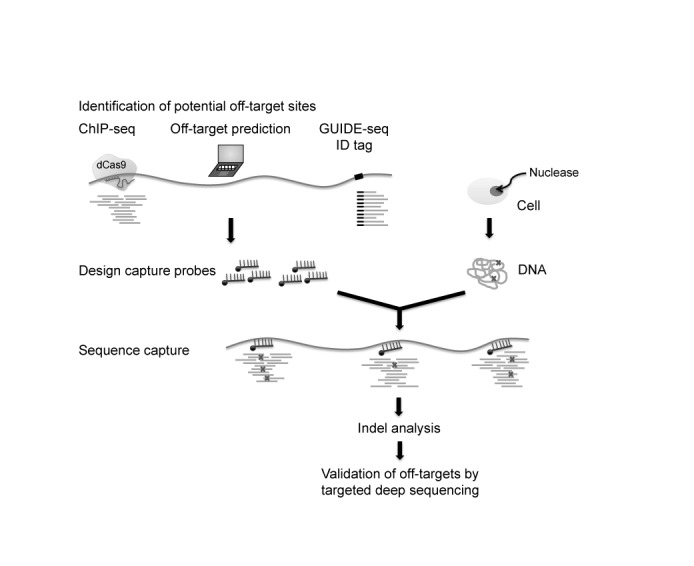
Schematic view of target capture as a comprehensive screen for nuclease-induced indels. Potential nuclease off-target sites are identified using a variety of sources such as GUIDE-seq, ChIP-seq and computational prediction. Regions selected by any or all of these methods are used to design capture probes. These probes are then used to enrich for genomic regions spanning potential off-target sites using DNA from cells treated with nuclease of choice. Indel analysis by high-throughput sequencing identifies *bona fide* indels within detection limits of the methodology. Identified off-target sites could further be validated by targeted amplicon sequencing.

As CRISPR-Cas9 sweeps across the field of biological sciences, creative experiments are being developed to investigate off-target sites. In addition to ChIP-seq and computational off-target tools, GUIDE-seq is an alternative approach to detect off-target cleavage on a genome-wide scale (54). Sequence capture allows for cost-effective and high-throughput screening of off-target sites identified by any method (Figure 7) and can be used to determine off-target activity of not only RNA-guided nucleases, but also other genome editing tools such as zinc finger nucleases and TALENs. We can then use this knowledge to design and choose more target-specific genome editing tools.

## SUPPLEMENTARY DATA

Supplementary Data are available at NAR Online.

SUPPLEMENTARY DATA
