# Supplementary material for: A genome-wide analysis of Cas9 binding specificity using ChIP-seq and targeted sequence capture
Source: Nucleic Acids Res. 2015 Feb 20;43(6):3389–404. doi: 10.1093/nar/gkv137 (PMC4381059; doi:10.1093/nar/gkv137)
Supplement: SUPPLEMENTARY DATA [file supp_43_6_3389__index.html]

A genome-wide analysis of Cas9 binding specificity using ChIP-seq and targeted sequence capture — SUPPLEMENTARY DATA 

# A genome-wide analysis of Cas9 binding specificity using ChIP-seq and targeted sequence capture

## SUPPLEMENTARY DATA

**Files in this Data Supplement:**

- Supplementary Figures
- Supplementary Material
- Table S1
- Table S2
- Table S3
- Table S4
