# Supplementary material for: A genome-wide analysis of Cas9 binding specificity using ChIP-seq and targeted sequence capture
Source: Nucleic Acids Res. 2015 Feb 20;43(6):3389–404. doi: 10.1093/nar/gkv137 (PMC4381059; doi:10.1093/nar/gkv137)
Supplement: SUPPLEMENTARY DATA [file supp_gkv137_nar-03286-h-2014-File010.pdf]

O’Geen, Figure S1

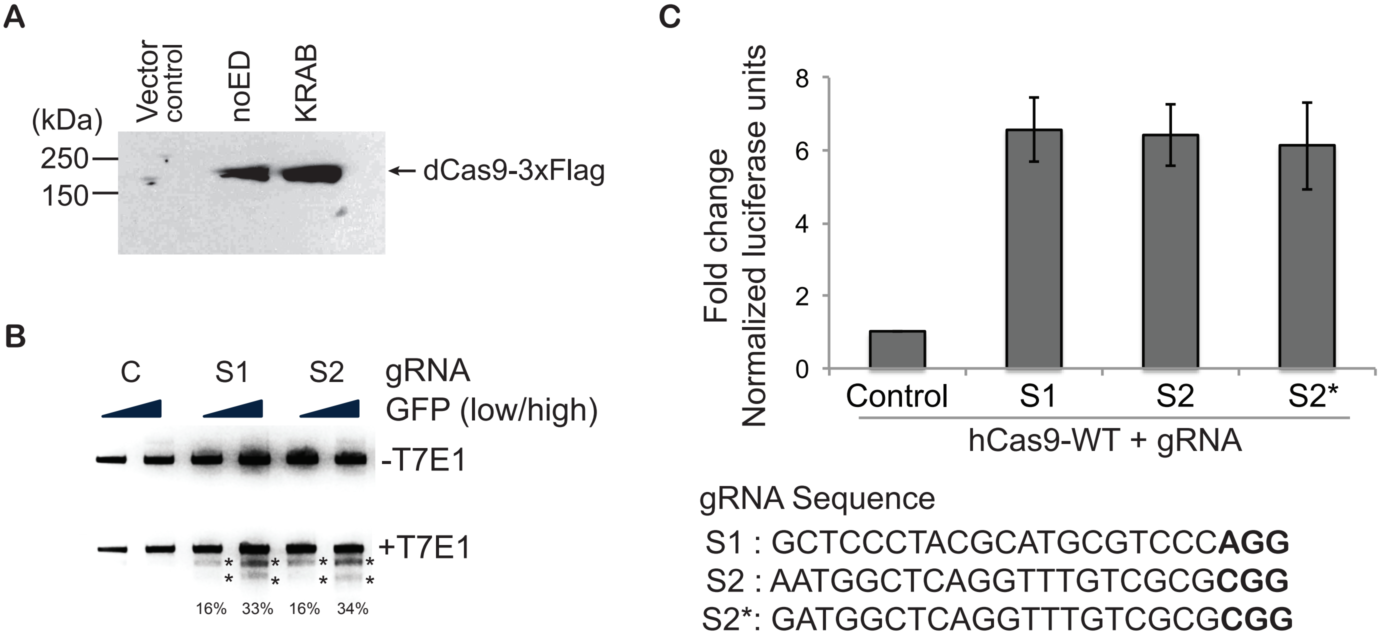

Figure S1. Evaluation of gRNA efficiency and dCas9 protein expression. (A) Western blot analysis of FLAG-tagged dCas9 protein containing either no effector domain (noED) or the KRAB domain. Whole-cell extracts from Neuro-2A cells transfected with the indicated FLAG-tagged plasmids or empty vector were blotted and probed with anti-FLAG-HRP antibody (SIGMA A8592). **(B)** T7E1 assay was used to confirm the genome-editing activity of targeted Cas9 in Neuro-2A cells. Cells were transfected with hCas9-WT, a GFP-expressing plasmid (pCMV-eGFP) and S1 gRNA, S2 gRNA or a non-functional gRNA control C. Cells were sorted into low and high GFP expressing cells. Genomic DNA was isolated and a ~800 bp PCR product was amplified using primers flanking the target site (Supplemental Table S1). Amplicons were heat denatured and slowly re-annealed to allow formation of heteroduplexes that are cleaved by T7 endonuclease I releasing two smaller DNA fragments (marked with asterisks). DNA fragments were quantitated using the Gel Doc XR system (BioRad) and indel frequency was calculated. Calculated indel frequencies are shown below samples treated with T7E1 enzyme. **(C)** The Single-Strand Annealing (SSA) recombination reporter assay was used to determine RNA guided Cas9 cleavage activity as previously described (Bhakta et al. 2013). S1, S2 or S2* gRNA target sites were introduced into the split firefly luciferase gene. Targeted Cas9 cleavage activity results in joining of the split luciferase gene. Firefly luciferase activity was determined and normalized to a Renilla luciferase control signal. All experiments were performed in duplicates.

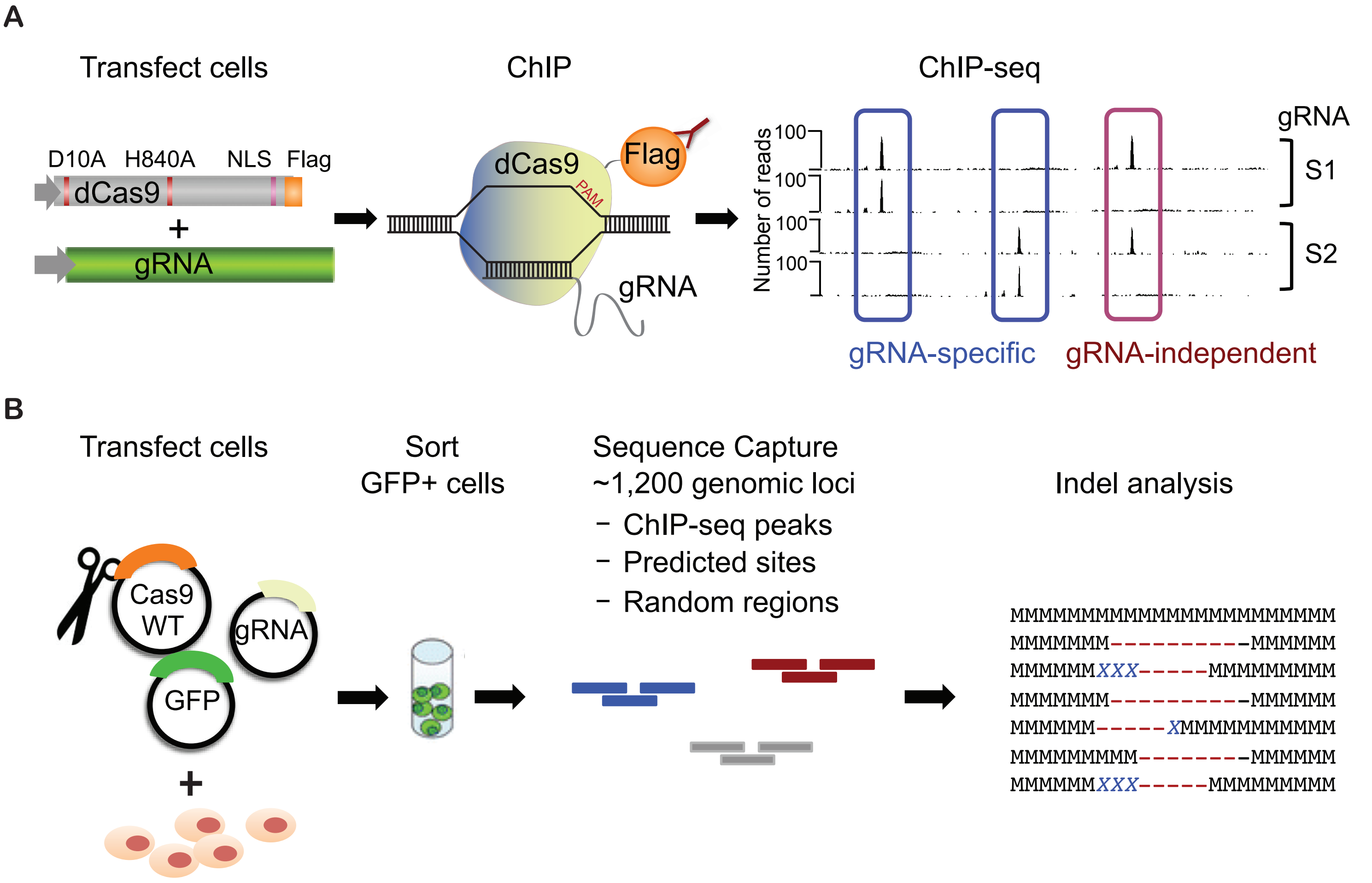

Figure S2. Experimental design for genome-wide Cas9 binding and activity analysis. (A) Schematic of ChIP-seq analysis to identify gRNA-specific and gRNA-independent binding sites. **(B)** Schematic of sequence capture to evaluate Cas9 cleavage activity at potential off-target sites identified by ChIP-seq and computational prediction based on sequence similarity to target site.

O’Geen, Figure S3

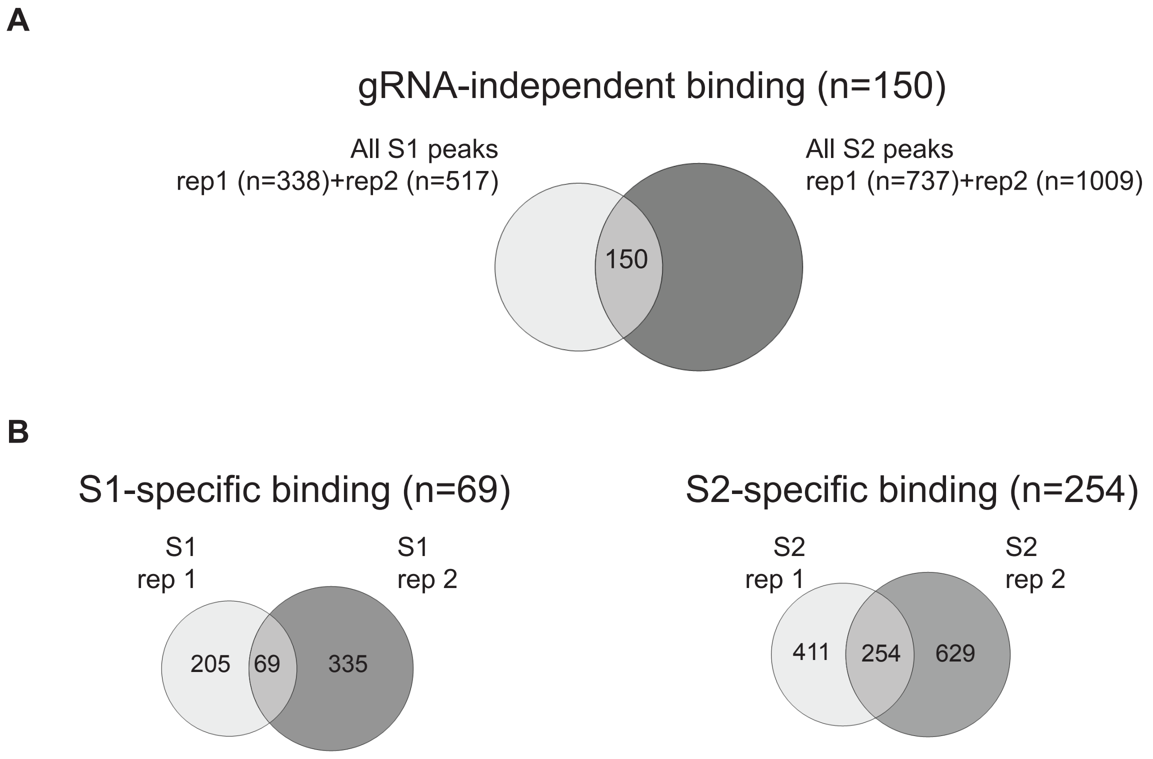

Figure S3. Identification of S1 and S2 specific Cas9 binding sites. Venn diagrams showing the overlap between ChIP-seq data sets. **(A)** S1 peaks from two independent experiments (rep1 and rep2) were overlapped with S2 peaks from two independent experiments. The 150 overlapping peaks were considered as gRNA-independent since they were observed with two different gRNAs (S1 and S2 respectively). **(B)** gRNA-independent peaks present in S1 and S2 gRNA data sets were subtracted from S1 and S2 gRNA-specific binding peaks prior to overlap analysis. Overlaps identified 69 and 254 (S1 and S2 respectively) *bona fide* binding sites for downstream analysis.

Figure S4: Amino acid sequence of dCas9-KRAB-3X Flag
5-Amino acid linker is underlined
KRAB domain is highlighted in blue
3XFlag tag is highlighted in orange

1 " DKKYSIGLD IGTNSVGWAV ITDEYKVPSK KFKVLGNTDR HSIKKNLIGA
51 LLFDSGETAE ATRLKRTARR RYTRRKNRIC YLQEIFSNEM AKVDDSFFHR
101 LEESFLVEED KKHERHPIFG NIVDEVAYHE KYPTIYHLRK KLVDSTDKAD
151 LRLIYLALAH MIKFRGHFLI EGDLNPDNSD VDKLFIQLVQ TYNQLFEENP
201 INASGVDAKA ILSARLSKSR RLENLIAQLP GEKKNGLFGN LIALSLGLTP
251 NFKSNFDLAE DAKLQLSKDT YDDDLDNLLA QIGDQYADLF LAAKNLSDAI
301 LLSDILRVNT EITKAPLSAS MIKRYDEHHQ DLTLLKALVR QQLPEKYKEI
351 FFDQSKNGYA GYIDGGASQE EFYKFIKPIL EKMDGTEELL VKLNREDLLR
401 KQRTFDNGSI PHQIHLGELH AILRRQEDFY PFLKDNREKI EKILTFRIPY
451 YVGPLARGNS RFAWMTRKSE ETITPWNFEE VVDKGASAQS FIERMTNFDK
501 NLPNEKVLPK HSLLYEYFTV YNELTKVKYV TEGMRKPAFL SGEQKKAIVD
551 LLFKTNRKVT VKQLKEDYFK KIECFDSVEI SGVEDRFNAS LGTYHDLLKI
601 IKDKDFLDNE ENEDILEDIV LTLTLFEDRE MIEERLKTYA HLFDDKVMKQ
651 LKRRRYTGWG RLSRKLINGI RDKQSGKTIL DFLKSDGFAN RNFMQLIHDD
701 SLTFKEDIQK AQVSGQGDSL HEHIANLAGS PAIKKGILQT VKVVDELVKV
751 MGRHKPENIV IEMARENQTT QKGQKNSRER MKRIEEGIKE LGSQILKEHP
801 VENTQLQNEK LYLYYLQNGR DMYVDQELDI NRLSDYDVDA IVPQSFLKDD
851 SIDNKVLTRS DKNRGKSDNV PSEEVVKKMK NYWRQLLNAK LITQRKFDNL
901 TKAERGGLSE LDKAGFIKRQ LVETRQITKH VAQILDSRMN TKYDENDKLI
951 REVKVITLKS KLVSDFRKDF QFYKVREINN YHHAHDAYLN AVVGTALIKK
1001 YPKLESEFVY GDYKVYDVRK MIAKSEQEIG KATAKYFFYS NIMNFFKTEI
1051 TLANGEIRKR PLIETNGETG EIVWDKGRDF ATVRKVLSMP QVNIVKKTEV
1101 QTGGFSKESI LPKRNSDKLI ARKKDWDPKK YGGFDSPTVA YSVLVVAKVE
1151 KGKSKKLKSV KELLGITIME RSSFEKNPID FLEAKGYKEV KKDLIIKLPK
1201 YSLFELENGR KRMLASAGEL QKGNELALPS KYVNFLYLAS HYEKLKGSPE
1251 DNEQKQLFVE QHKHYLDEII EQISEFSKRV ILADANLDKV LSAYNKHRDK
1301 PIREQAENII HLFTLTNLGA PAAFKYFDTT IDRKRYTSTK EVLDATLIHQ
1351 SITGLYETRI DLSQLGGDSR ADPKKKRKVA AASGGGGS**VT** **FKDVFVDFTR**
1401 **EEWKLLDTAQ** **QIVYRNV! LE** **NYKNLVSLGY** **QLTKPDVILR** **LEKGEEPWLV** !
1451 **EREIHQETHP** AS**DYKDHDGD** **YKDHDIDYKD** **DDDK***

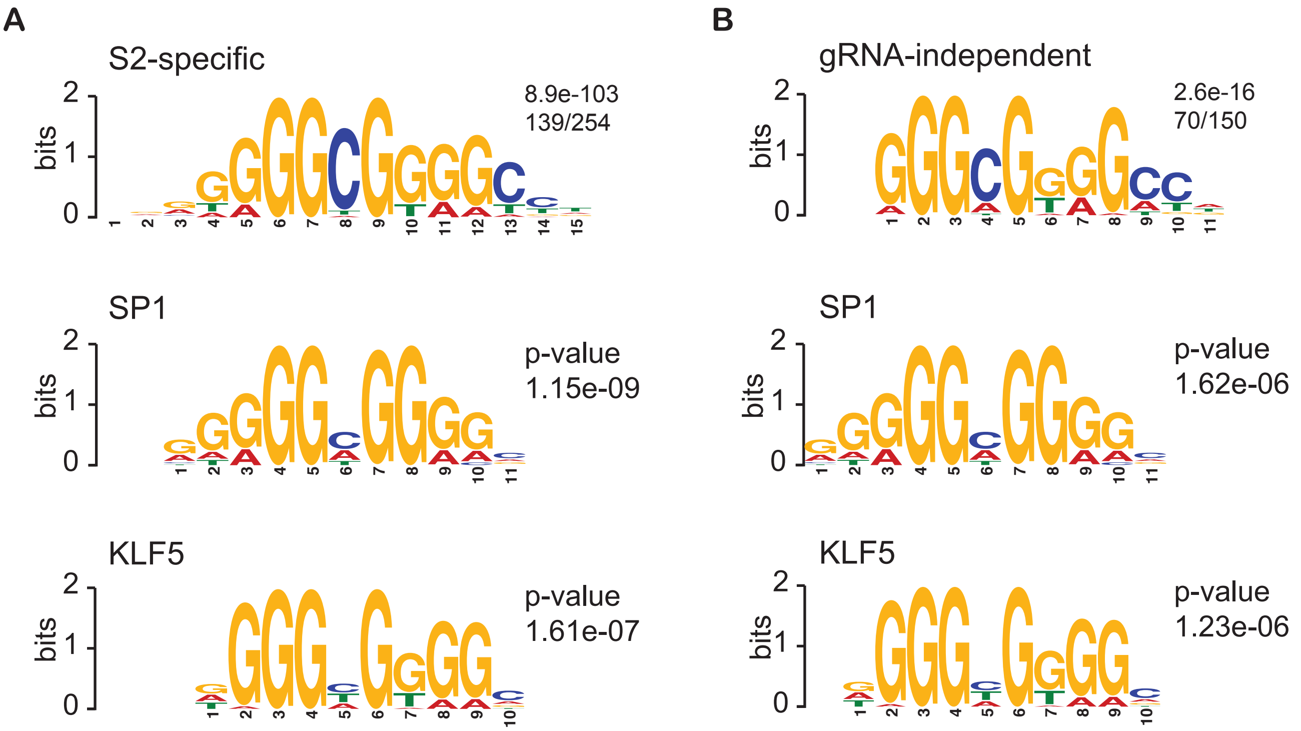

Figure S5. S2-specific and gRNA-independent motifs resemble SP1 and KLF5 motifs. The Tomtom Motif Comparison Tool identified SP1 and KLF5 motifs when S2-specific **(A)** and gRNA-independent **(B)** motifs are compared with the elements of a database of known motifs (and their DNA reverse complements).

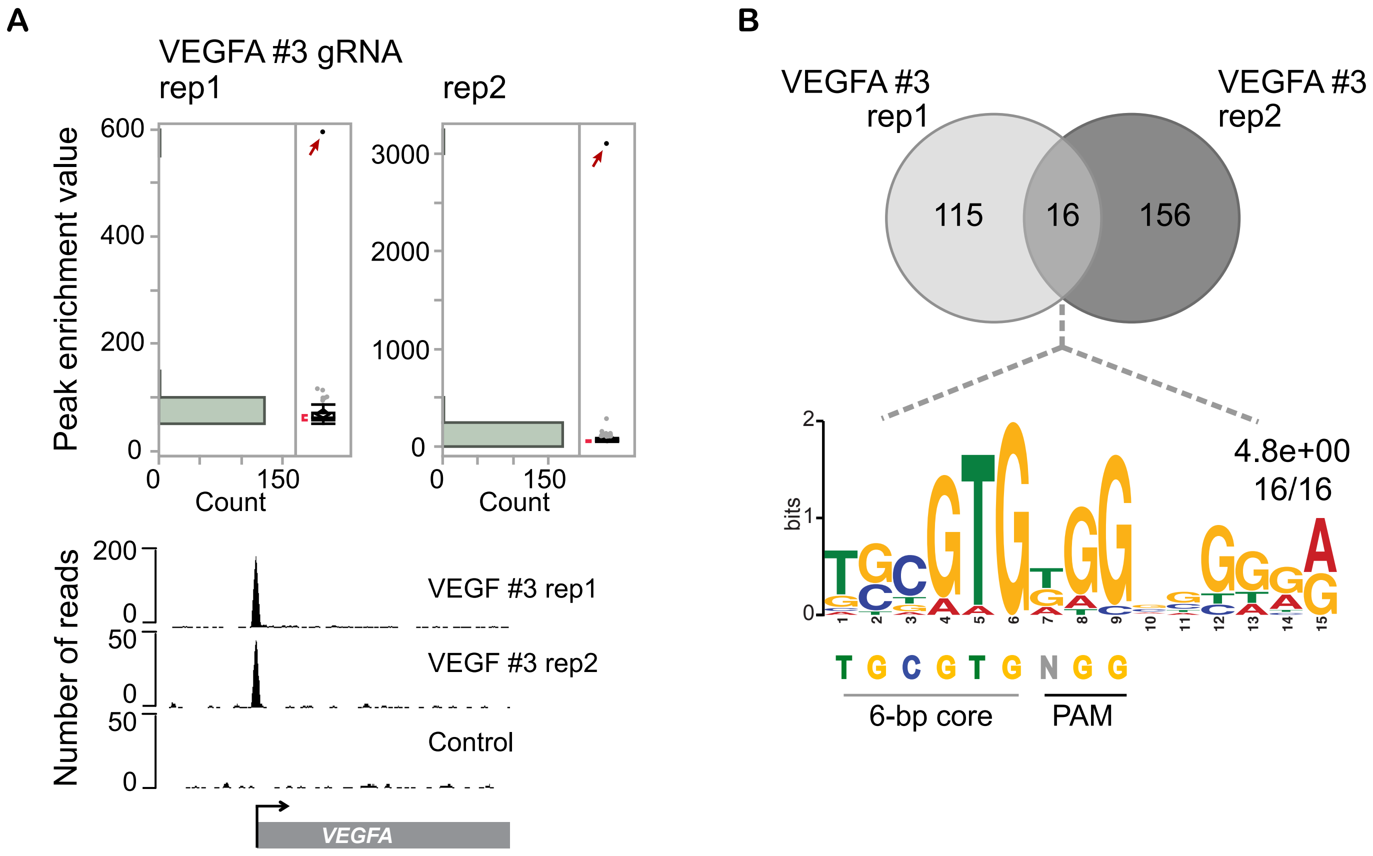

Figure S6. Genome-wide Cas9 binding in human cell line HEK293T facilitated by *VEGFA* #3 gRNA. (A) HEK293T cells were co-transfected with the Flag-tagged dCas9 expressing plasmid and a plasmid expressing *VEGFA* #3 gRNA or a non-functional gRNA (Control). ChIP-seq peak distribution in two independent biological replicates determines that ChIP-seq enrichment is highest at the target site (marked with arrow). dCas9-binding profile shows ChIP-enrichment at the human *VEGFA* locus. No enrichment was detected in the Control data set. **(B)** Overlap analysis of Cas9-bound regions reveals 16 sites in common between two biological replicates. MEME-ChIP de novo motif analysis identifies a 6-bp core motif and extends beyond the PAM.

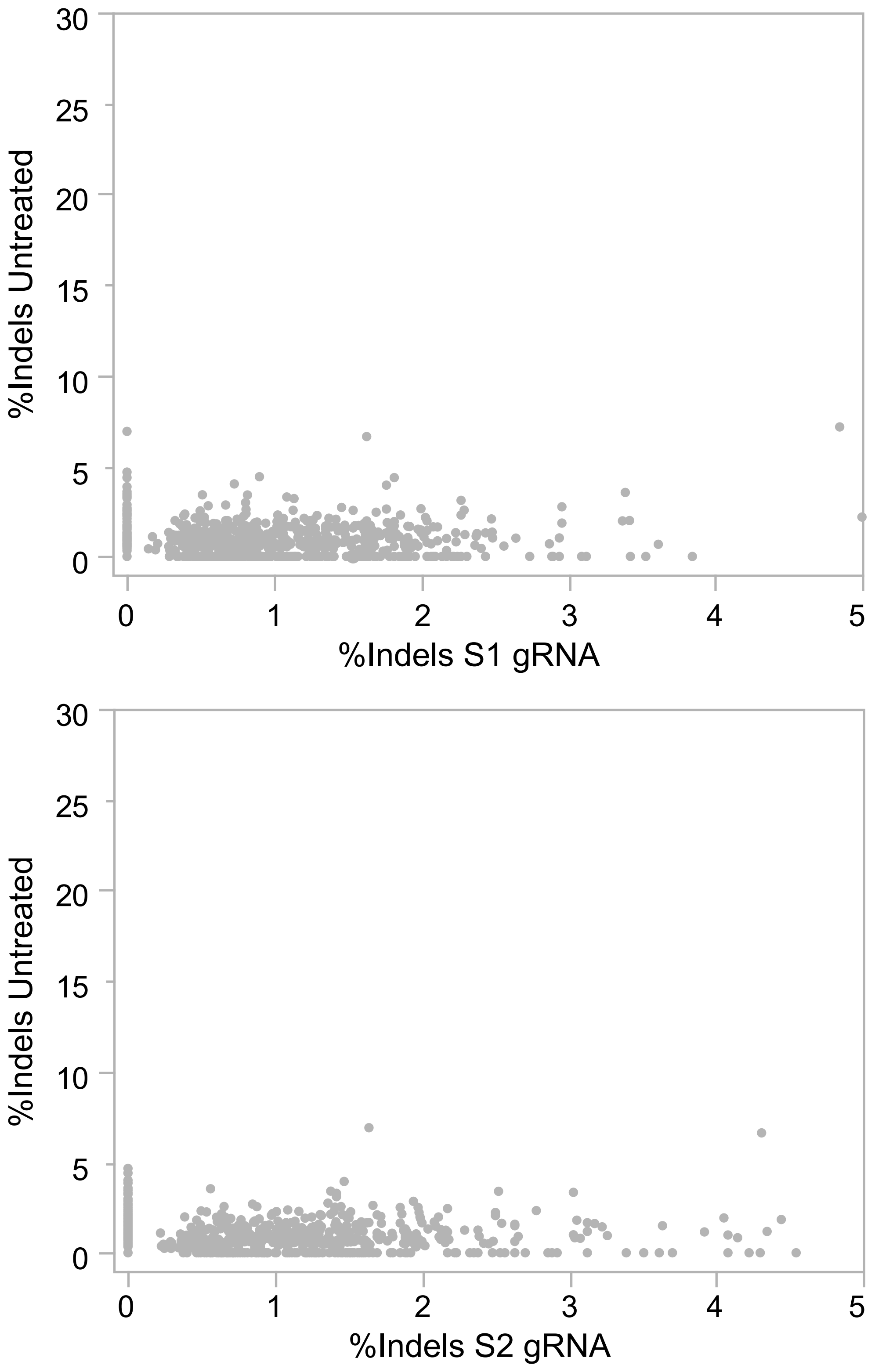

Figure S7. Sequence capture does not identify significant indels in untreated Neuro-2A samples. Bivariate analysis of percent indels relative to the total number of reads per target region. Neuro-2a cells expressing catalytically active Cas9 and one of three gRNA variations are compared. The top panel depicts a comparison of cells expressing non-functional gRNA to cells treated with S1 gRNA, while the bottom compares non-functional gRNA expressing cells against cells treated with S2 gRNA. No significant indels were identified by Fisher's exact test ($n=1200$, $p<0.01$). S1 or S2 gRNA-treated samples that had less than 5% indel frequency were compared to non-functional gRNA control sample.

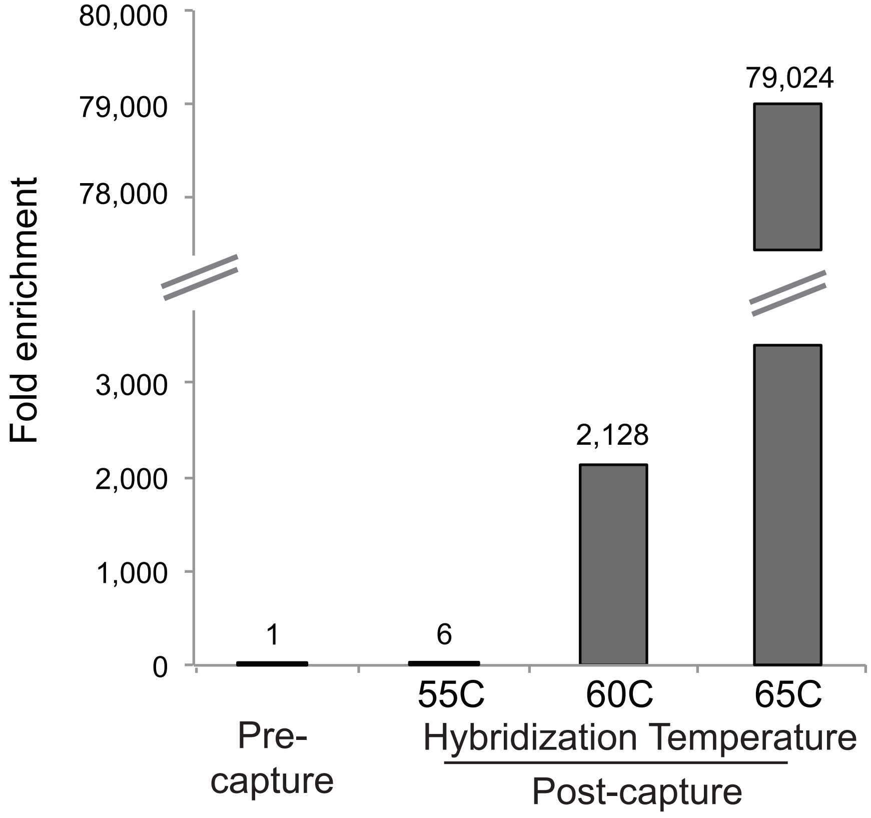

Figure S8. Validation of capture enrichment. Capture enrichment was determined using quantitative real-time PCR. Fold enrichment was calculated using the 2^-ddCT method comparing the S1/S2 target site to a promoter region not present on the capture baits. No enrichment was determined before targeted sequence capture. Capture efficiency was monitored for three different hybridization temperatures showing strongest enrichment at 65C.

Sample	Indel% TS	Indel% OT1
empty gRNA	0.2	0.1
S1 gRNA	33.5 *	0.0
S2 gRNA	34.7 *	16.0 *

Figure S9. Indel analysis by targeted high-throughput sequencing.
Results summarize the percentage of sequencing reads containing indels in Neuro-2a cells treated with Cas9 nuclease together with either S1, S2 or empty gRNA. Indel percentages were determined for the S1 and S2 target sites as well as the S2 off-target site OT1. Asterisk (*) indicates samples with statistically significant indels as compared to empty gRNA control samples (P<0.01, Fisher exact test).

A

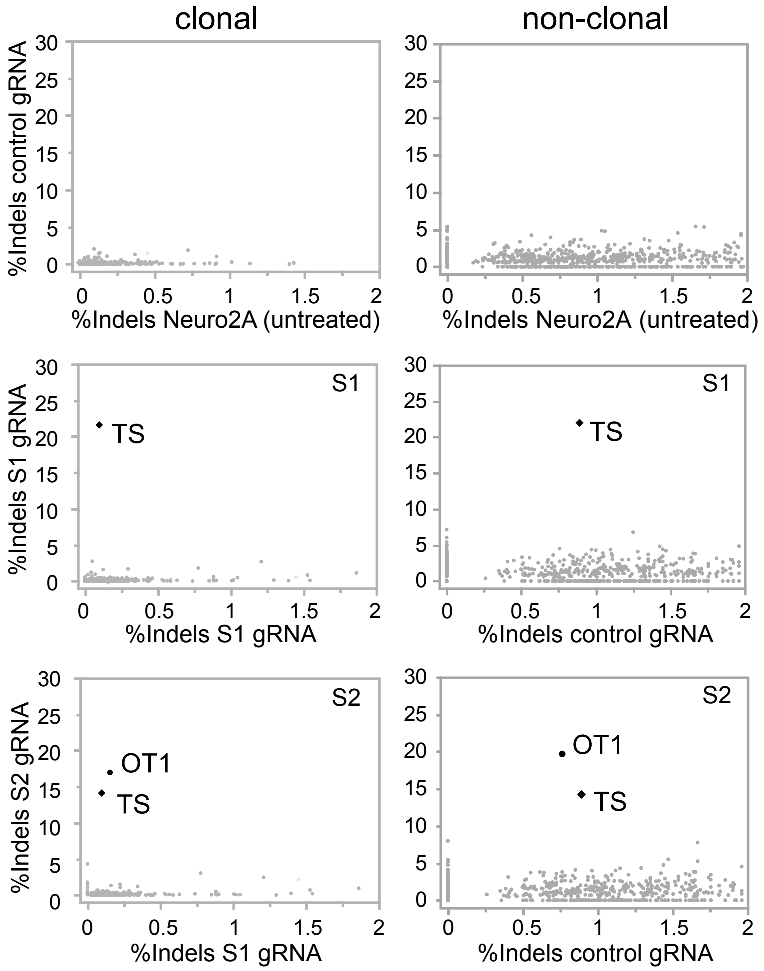

B

	% Indel			
	Neuro-2a	Empty gRNA	S1 gRNA	S2 gRNA
clonal				
TS	0.1	0.1	22	14.2
S2 OT1	0	0.2	0.1	17
non-clonal				
TS	0	0.9	22	14.3
S2 OT1	0	0.8	1.2	19.7

Figure S10. Sequence capture analysis using clonal and non-clonal reads.
(A) Bivariate analysis of percent indels relative to the total number of reads per target region. Indel analysis was performed two ways: (1) with all sequence reads passing quality filtering (clonal, left panel) and (2) with only unique reads after clonal sequences have been removed (non-clonal, right panel). The top panel depicts a comparison of Neuro-2a control cells expressing catalytically active Cas9 and non-functional gRNA to untreated Neuro-2a. The middle panel shows a comparison of cells treated with S1 gRNA against control cells. High occurrence of indels is observed for the target site (TS). The bottom panel shows a comparison of cells treated with S2 gRNA to control cells expressing non-functional gRNA. High indel frequencies are observed for one off-target site (OT1) in addition to the target site (TS). (B) A comparison of S2 TS and OT1 sequences, and a table listing the percentage of indels calculated for each of the four data sets at S1 and S2 TS and S2 OT1 for analysis using all reads (clonal) or after removal of clonal reads (non-clonal).
