# Supplementary material for: A genome-wide analysis of Cas9 binding specificity using ChIP-seq and targeted sequence capture
Source: Nucleic Acids Res. 2015 Feb 20;43(6):3389–404. doi: 10.1093/nar/gkv137 (PMC4381059; doi:10.1093/nar/gkv137)
Supplement: SUPPLEMENTARY DATA [file supp_gkv137_nar-03286-h-2014-File009.docx]

**Supplementary material for:**

**A genome-wide analysis of Cas9 binding specificity using ChIP-seq and targeted sequence capture**

Henriette O’Geen,^1^ Isabelle M. Henry,^2^ Mital S. Bhakta,^1^ Joshua F. Meckler,^1^ and David J. Segal^1,^ *

^1^ Genome Center and Department of Biochemistry and Molecular Medicine, University of California, Davis, CA, 95616, USA

^2^ Department of Plant Biology and Genome Center, University of California, Davis, CA, 95616, USA

* To whom correspondence should be addressed. Tel: 530-754-9134; Fax: 530-754-9658; Email: djsegal@ucdavis.edu

Supplemental Figure S1. Evaluation of gRNA efficiency and dCas9 protein expression.

Supplemental Figure S2. Experimental design for genome-wide Cas9 binding and activity analysis.

Supplemental Figure S3. Identification of S1 and S2 specific Cas9 binding sites.

Supplemental Figure S4. Amino acid sequence of dCas9-KRAB-3X Flag.

Supplemental Figure S5. S2-specific and gRNA-independent motifs resemble SP1 and KLF5 motifs.

Supplemental Figure S6. Genome-wide Cas9 binding in human cell line HEK293T facilitated by *VEGFA* #3 gRNA.

Supplemental Figure S7. Sequence capture does not identify significant indels in control samples.

Supplemental Figure S8. Validation of capture enrichment.

Supplemental Figure S9. Indel analysis by targeted high-throughput sequencing.

Supplemental Figure S10. Sequence capture analysis using clonal and non-clonal reads.

Supplemental Table S1. List of oligonucleotide sequences.

Supplemental Table S2. Summary of ChIP-seq results.

Supplemental Table S3. Location analysis of ChIP-seq peaks at S1, S2 or of gRNA-independent peaks in Neuro-2a cells.

Supplemental Table S4. Identification of Cas9-bound sites with up to four mismatches to the 12-bp seed region of either S1 or S2 target sequence.
